# Metformin inhibits cervical cancer cell proliferation via decreased AMPK O-GlcNAcylation

**DOI:** 10.1080/19768354.2019.1614092

**Published:** 2019-05-14

**Authors:** Min Young Kim, Yoon Sook Kim, Minjun Kim, Mee Young Choi, Gu Seob Roh, Dong Hoon Lee, Hyun Joon Kim, Sang Soo Kang, Gyeong Jae Cho, Jeong Kyu Shin, Wan Sung Choi

**Affiliations:** Gyeongsang National University, Jinju, The Republic of Korea

**Keywords:** AMPK, O-GlcNAcylation, p21, p27, cervical cancer cells

## Abstract

Metformin is a widely used drug for the treatment of type 2 diabetes. Antidiabetic drugs are also known to influence cancer progression, as high glucose levels affect both cancer and diabetes. Metformin induces cell cycle arrest in cancer cells, but the underlying mechanism remains unclear in cervical cancer system. Here, we examined how metformin affects cell cycle arrest and apoptosis in cervical cancer cells. Western blot analysis showed that levels of O-linked N-acetylglucosamine (O-GlcNAc) and O-GlcNAc transferase (OGT) were increased in cervical cancer cells; these effects were reversed by metformin treatment. Immunoprecipitation analysis was used to examine the interplay between O-GlcNAcylation and phosphorylation in HeLa cells, revealing that metformin decreased O-GlcNAcylated AMP-activated protein kinase (AMPK) and increased levels of phospho-AMPK compared to untreated cells. These results were associated with decreased cell cycle arrest and apoptotic cell death in HeLa cells, as shown by flow cytometry. Moreover, 6-diazo-5-oxo-L-norleucine (a glutamine fructose-6-phosphate aminotransferase inhibitor) or thiamet G (an O-GlcNAcase inhibitor) decreased or increased levels of O-GlcNAcylated AMPK, and increased or decreased levels of phosphorylated AMPK, respectively, suggesting that O-GlcNAc modification affects AMPK activation. Of note, we found that metformin treatment of HeLa cells increased the levels of p21 and p27 (which are AMPK-dependent cell cycle inhibitors), leading to increased cell cycle arrest and apoptosis in HeLa cells compared to untreated cells. These findings suggest that metformin may serve as a useful antiproliferative drug in cervical cancer cells, with potential therapeutic benefit.

## 1. Introduction

Cervical cancer is the fourth most frequently diagnosed cancer and the fourth leading cause of cancer deaths worldwide among females (Bray et al. 2018). Current treatment options are insufficient, and additional effective therapeutic strategies are needed.

Diverse molecular targets have been identified for which approved drugs already exist, and the potential repositioning of these drugs to new indications can be investigated (Li and Jones 2012). Repositioning or repurposing of a current therapeutic is an accelerated route for drug discovery because existing drugs have established clinical and pharmacokinetic data and are less expensive to develop (Li and Jones 2012).

Metformin is the first-line treatment for type 2 diabetes mellitus, showing established efficacy coupled with favourable safety and low cost, and therefore could be repositioned as an excellent anticancer drug candidate (Heckman-Stoddard et al. 2017).

The condition of hyperglycaemia present in diabetes mellitus may play a role in cancer development (Richardson and Pollack 2005). Many studies have shown an association between metformin treatment and decreased cancer risk (Rizos and Elisaf 2013), establishing metformin as a potential anticancer drug.

Metformin’s mechanism of action is via a glucose-lowering effect, which includes the activation of the energy sensor AMP-activated protein kinase (AMPK) (Foretz et al. 2014), which has been associated with the inhibition of glucose production in primary hepatocytes (Zhou et al. 2001). A previous study showed that metformin has an antiproliferative effect associated with cell cycle arrest, which is mediated by AMPK activation (Queiroz et al. 2014): Metformin was shown to modulate the expression of the cell cycle inhibitors p21 and p27 to arrest the cell cycle at the G0/G1 boundary, which may be due to AMPK activation (Cai et al. 2013). However, the mechanism by which metformin affects AMPK activation related to p21 and p27 modulation remains unclear.

Cancer cells take up large amounts of glucose and glutamine from their environment, and in response to oncogene activation, cancer cells experience elevated flux via the hexosamine biosynthetic pathway (HBP) (Ferrer et al. 2016). Oncogenic RAS increases this flux via both glycolysis and the HBP, resulting in increased O-GlcNAcylation (Ferrer et al. 2016). UDP-GlcNAc serves as a donor substrate for O-GlcNAc transferase (OGT)-mediated O-GlcNAcylation of a number of nuclear and cytoplasmic proteins; UDP-GlcNAc can be covalently added onto serine and threonine residues of a wide range of cellular proteins solely through the action of OGT (Bond and Hanover 2015). This modification can be removed by the glycoside hydrolase O-GlcNAcase (OGA) that catalyzes the cleavage of O-GlcNAc from proteins (Bond and Hanover 2015). Many studies have indicated that increased O-GlcNAcylation is a general feature of cancer and contributes to the transformed phenotype (Ma and Vosseller 2014). In a previous study, we found that the levels of O-GlcNAcylation were elevated in cervical cancer cells (Kim et al. 2016).

The cross-talk between O-GlcNAcylation and phosphorylation has been shown to regulate the activation of AMPK and alter the substrate selectivity of OGT in several cell lines (Bullen et al. 2014), potentially affecting cellular gene expression, cell growth, and apoptotic cell death (Hart et al. 2011).

In this study, we examined whether metformin affects AMPK O-GlcNAcylation, which may then alter the levels of p21 and p27, and subsequently influence cell cycle arrest and apoptotic cell death via its inhibitory effects on cell proliferation.

## 2. Materials and methods

### 2.1. Cell lines

The HeLa cervical cancer cell line [High-risk Human Papillomavirus (HPV)-18-positive] and the HaCaT human keratinocyte cell line (HPV-negative cell line) were purchased from the American Type Culture Collection (Manassas, VA, USA). HPVs encode E6 and E7 oncoproteins, which promote cervical cancer. Therefore, we used HaCaT cells as control cells to suggest anti-cancer effects of metformin in cervical cancer cell line (HeLa cells). HeLa and HaCaT cells were maintained in Dulbecco’s modified Eagle’s medium and minimum essential media in 5% CO_2_ at 37°C. All media were supplemented with 10% foetal bovine serum (Invitrogen, Carlsbad, CA, USA), 100 μg/mL streptomycin, and 100 units/mL penicillin (Invitrogen).

### 2.2. Cell proliferation assay

HeLa and HaCaT cells were seeded at a density of 1.0 × 10^4^ cells/well in a 96-well plate, and a 3-(4,5-dimethylthiazol-2-yl)-2,5-diphenyltetrazolium (MTT) (Sigma, St. Louis, MO, USA) assay was conducted after a 24-h metformin treatment. Metformin concentrations of 0, 10, 20, 40, 80, and 100 mM were used. The MTT solution (2 mg/mL) was added to each well, and the plates were incubated at 37°C for 2 h. The resulting formazan crystals were dissolved in dimethyl sulfoxide, and the absorbance of the solution was measured at 570 nm using a microplate reader (Tecan, Maennedorf, Switzerland).

### 2.3. Analyses of the cell cycle and apoptosis

Cells were collected and seeded into six-well plates at 1 × 10^5^ cells per well and cultured for 16 h. The cells were synchronized by serum deprivation and then treated with 50 mM metformin for 24 h, digested with trypsin, fixed with cold 90% ethanol, and incubated for 1 h at 4°C. Cells were pelleted and resuspended in 1 mL PBS containing propidium iodide (1 mg/mL) and RNase A (1 mg/mL). Following incubation at 37°C for 30 min, analyses of the cell cycle and apoptosis (sub-G1 phase) were performed by flow cytometry (FACscan, BD Biosciences, San Jose, CA, USA), according to the manufacturer’s protocol, using CXP 2.2 software. Experiments were repeated three times separately.

### 2.4. Detection of apoptotic cells by annexin-V-FITC/propidium iodide (PI) double staining

Apoptosis was analyzed using fluorescence-activated cell sorting with annexin-V-FITC and PI double staining. Cells were collected and seeded into six-well plates at 1 × 10^5^ cells per well and cultured for 24 h. Cells were then treated with 50 mM metformin for 24 h, digested with trypsin, fixed with cold 90% ethanol, and incubated for 1 h at 4°C. Cells were pelleted and resuspended in binding buffer (0.1 M Hepes, pH 7.4, 1.4 M NaCl, and 25 mM CaCl2 solution) for 15 min at room temperature in the dark. Apoptotic cells were detected by flow cytometry. Analyses were performed using CXP 2.2 software. Experiments were repeated three times separately.

### 2.5. Western blot analysis

Cells were homogenized in lysis buffer (50 mM Tris, pH 7.5, 150 mM NaCl, 5 mM EDTA, and 1% Nonidet P-40) and protease inhibitor cocktail (Sigma). Total proteins (10–20 μg) were separated by SDS-PAGE on 10% acrylamide gels and transferred onto nitrocellulose membranes (Millipore, Billerica, MA, USA). The membranes were probed with the following primary antibodies: O-GlcNAc (RL2) (1:10,000; Thermo Fisher Scientific Inc., Rockford, IL, USA), OGT (1:5,000; Santa Cruz Biotechnology, CA, USA), AMPK*α* (1:5,000), *p*-AMPK*α* (1:5,000; Cell Signaling), p21 (1:5,000; Santa Cruz Biotechnology), p27 (1:5,000; Santa Cruz Biotechnology), poly(ADP-ribose) polymerase (PARP, 1:5,000; Cell Signaling #9532), cleaved PARP (1:5,000; Cell Signaling #5625), and *β*-actin (1:10,000; PIERCE, Rockford, IL, USA). Immunoreactive antigens were detected using the Enhanced Chemiluminescence Detection kit (ECL, Amersham Bioscience, Piscataway, NJ, USA). The specific protein bands were analyzed by densitometry, and the values were normalized to that of the *β*-actin control and further analyzed using Image J software (Bethesda, MD, USA).

### 2.6. Succinylated wheat germ agglutinin (sWGA) affinity purification

HeLa and HaCaT cells were lysed with RIPA lysis buffer (150 mM NaCl, 50 mM Tris, pH 7.4, 1 mM EDTA, 0.5% Nonidet P-40), and the cell lysates (∼200 µg protein) were incubated with agarose-conjugated sWGA beads (Vector Laboratories, Burlingame, CA, USA) overnight at 4°C. For the control, the inhibitory monosaccharide GlcNAc was added during the sWGA-lectin-affinity purification. Precipitates were washed three times with lysis buffer, and the proteins were eluted by boiling in SDS sample buffer.

### 2.7. Immunoprecipitation (IP)

Protein extracts were mixed with protein A/G agarose beads (Santa Cruz Biotechnology), incubated for 1 h at 4°C, and then centrifuged at 5,000 × *g* for 1 min. The supernatant was incubated with the IP antibodies overnight at 4°C and then incubated with protein A/G agarose beads for 2 h at 4°C. The negative control was prepared using protein A/G agarose beads without the antibody. The protein-bead complex was then washed and collected by centrifugation, samples were boiled in loading buffer to remove the agarose beads, and the protein (2 mg) was then separated by SDS-PAGE on 10% acrylamide gels. Proteins were then transferred onto membranes, probed with antibodies against the interacting protein of interest, and processed for western blotting, as described above.

### 2.8. Statistical analysis

Data are representative of three independent experiments and presented as the mean ± S.E.M. Statistical analyses were performed using the Student’s *t* test or ANOVA (GraphPad Prism, La Jolla, CA). *P* values less than 0.05 were considered statistically significant.

## 3. Results

### 3.1. Metformin inhibits cell proliferation and increases apoptosis in HeLa and HaCaT cells

**Figure 1.**
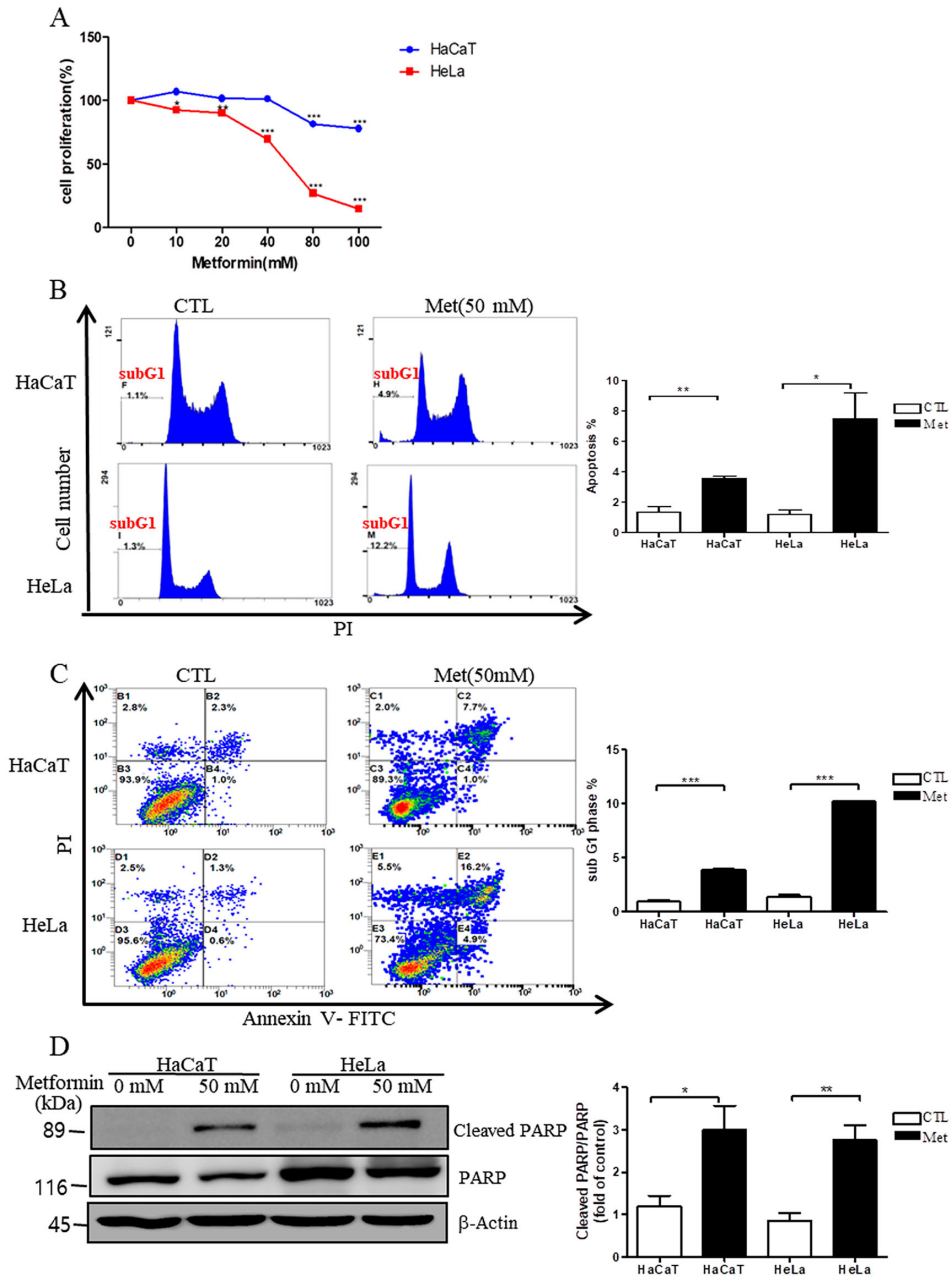
Metformin inhibits cell proliferation and increases apoptosis and levels of cleaved PARP in HeLa cells. Cell proliferation measured by MTT assay (A), and levels of control cells (HaCaT) and cervical cancer cells (HeLa) in sub-G1 phase (B) and apoptosis (C) with (Met 50 mM) or without (CTL) metformin treatment, as measured by flow cytometry. (D) Representative western blot and quantification of cleaved PARP in HaCaT or HeLa cells with (Met 50 mM) or without (CTL) metformin treatment. Band intensity was normalized to that of *β*-actin. Data are representative of three independent experiments and presented as the mean ± S.E.M. *P < 0.05, **P < 0.01, ***P < 0.001 vs. CTL.

To determine the effects of metformin on cell proliferation in HeLa and HaCaT cells, we carried out an MTT assay after the treatment of cells with different concentrations of metformin. We found that metformin inhibits cell proliferation in a concentration-dependent manner in HeLa and HaCaT cells (Figure 1(A), *P < 0.05, **P < 0.01 or ***P < 0.001) and that this inhibition was greater in HeLa cells than in HaCaT cells (Figure 1(A)). Further, to test whether metformin affects cell cycle arrest and apoptosis in HeLa and HaCaT cells, flow cytometry analyses were performed. We found that metformin treatment of HeLa cells significantly increased cell cycle arrest and apoptosis compared to HeLa cells without metformin (Figure 1(B and C), ***P < 0.001 and *P < 0.05, respectively). Consistently, western blot analysis showed that metformin treatment of HeLa cells significantly increased the levels of cleaved PARP, which is related to cell death, compared to HeLa cells without metformin (Figure 1(D), ***P < 0.001).

### 3.2. Metformin decreases levels of OGT and O-GlcNAc in HeLa cells

**Figure 2.**
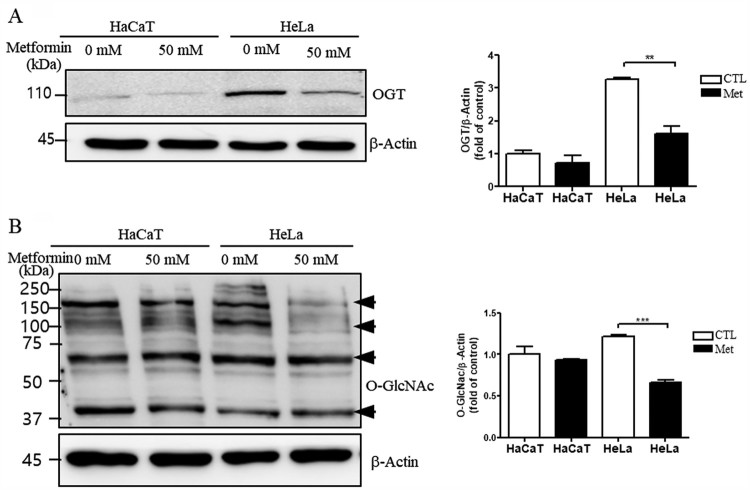
Metformin decreases the levels of OGT and O-GlcNAc in HeLa cells. Representative western blots and quantification of OGT (A) and O-GlcNAc (B) in HaCaT or HeLa cells with (Met 50 mM) or without (CTL) metformin treatment. Band intensity was normalized to that of *β*-actin. Data are representative of three independent experiments and presented as the mean ± S.E.M. **P < 0.01, ***P < 0.001 vs. CTL.

To test whether metformin affects OGT and O-GlcNAc, which may alter the levels of AMPK (Hart et al. 2011), we measured the levels of OGT and O-GlcNAc in HeLa and HaCaT cells. Western blot analysis showed that levels of OGT and O-GlcNAc were significantly decreased in HeLa cells treated with metformin compared to HeLa cells without metformin (Figure 2(A and B), **P < 0.01 and ***P < 0.001, respectively).

### 3.3. O-GlcNAcylation regulates the levels of AMPK activation, and metformin decreases AMPK O-GlcNAcylation and increases AMPK activation in HeLa cells

**Figure 3.**
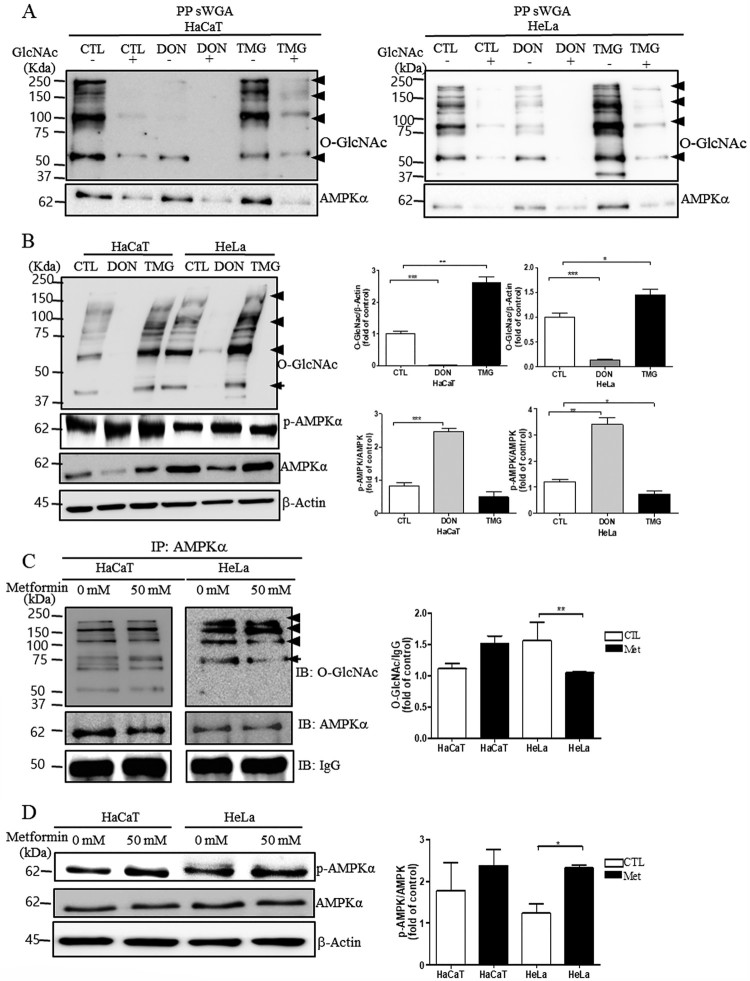
O-GlcNAc regulates the levels of AMPK activation, and metformin decreases AMPK interactions with O-GlcNAc and increases levels of phospho-AMPK in HeLa cells. (A) Representative WGA affinity purification in HaCaT or HeLa cells. (B) Representative western blots and quantification of phospho-AMPK or O-GlcNAc in HaCaT cells or HeLa cells with or without (CTL) DON or TMG treatment. Band intensity was normalized to that of *β*-actin. Data are presented as the mean ± S.E.M. (C) Binding of AMPK to O-GlcNAc. Representative immunoblots (IBs) and quantification of co-immunoprecipitated O-GlcNAc to AMPK in HaCaT or HeLa cells with (Met 50 mM) or without (CTL) metformin treatment. Cell lysates were subjected to IP with an anti-AMPK antibody and immunoblotted with an anti-O-GlcNAc antibody. The same blots were reprobed with the IP antibody to confirm levels of protein loading. Densitometry values of co-immunoprecipitated O-GlcNAc to AMPK was normalized to IgG. Data are presented as the mean ± S.E.M. (D) Representative western blot and quantification of phospho-AMPK in HaCaT or HeLa cells with (Met 50 mM) or without (CTL) metformin treatment. Band intensity was normalized to that of *β*-actin. Data are representative of three independent experiments and presented as the mean ± S.E.M. *P < 0.05, **P < 0.01, ***P < 0.001 vs. CTL.

Affinity purification using sWGA (to show whether the antibody was able to truly recognize the sugar modification) showed that when the inhibitory monosaccharide GlcNAc was added during sWGA-lectin-affinity purification, O-GlcNAc mostly disappeared (Figure 3(A)), confirming antibody specificity. Further, to investigate the role of O-GlcNAc modification in AMPK activation, we treated HeLa or HaCaT cells with thiamet G, a highly selective OGA inhibitor, to stimulate O-GlcNAcylation. Notably, thiamet G significantly decreased the levels of phospho-AMPK in HeLa cells (Figure 3(B), *P < 0.05). Moreover, treatment of HeLa or HaCaT cells with 6-diazo-5-oxo-L-norleucine (DON) significantly increased the levels of phospho-AMPK in HeLa cells, confirming the role of O-GlcNAc modification in AMPK activation (Figure 3(B), **P < 0.01). As such, AMPK O-GlcNAcylation may inhibit AMPK activation (Bullen et al. 2014), so we tested whether metformin affects AMPK activation by measuring the levels of O-GlcNAcylated AMPK by IP analysis. We found that the levels of O-GlcNAcylated AMPK were significantly decreased in HeLa cells treated with metformin compared to HeLa cells without metformin (Figure 3(C), **P < 0.01). Consistently, western blot analysis showed that levels of phospho-AMPK were significantly increased in HeLa cells treated with metformin compared to HeLa cells without metformin (Figure 3(D), *P < 0.05).

### 3.4. Metformin increases levels of p21 and p27 in HeLa cells

**Figure 4.**
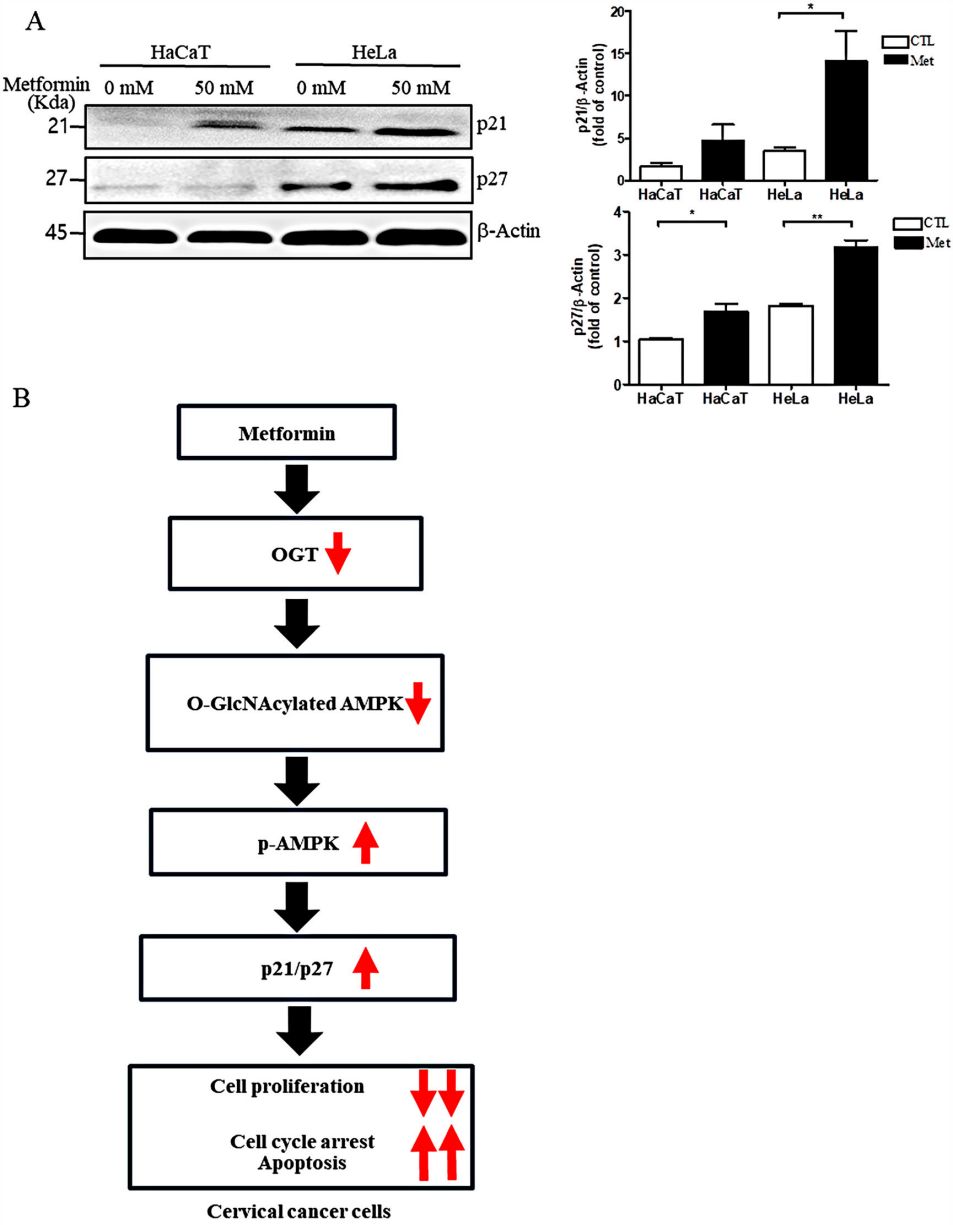
Metformin increases the levels of p21 and p27 in HeLa cells. (A) Representative western blots and quantification of p21 and p27 in HaCaT or HeLa cells with (Met 50 mM) or without (CTL) metformin treatment. Band intensity was normalized to that of *β*-actin. Data are representative of three independent experiments and presented as the mean ± S.E.M. *P < 0.05, **P < 0.01 vs. CTL. (B) Depiction of the signalling pathway involved in cell cycle arrest in cervical cancer cells after metformin treatment. Abbreviations: OGT, O-GlcNAc transferase; AMPK, AMP-activated protein kinase; p-AMPK, phospho-AMPK. Up- or down-facing arrows are the proposed increases or decreases in the levels of the respective molecular targets, respectively, after metformin treatment of cervical cancer cells.

We further tested whether metformin affects p21 and p27, which are AMPK-dependent cell cycle inhibitors (Pham et al. 2019), in HeLa cells. Western blot analysis showed that levels of p21 and p27 were significantly increased in HeLa cells treated with metformin compared to HeLa cells without metformin (Figure 4(A), *P < 0.05 and **P < 0.01, respectively), consistent with the metformin-induced cell cycle arrest in HeLa cells depicted in Figure 4(B).

## 4. Discussion

Metformin reduces the risk of cancer in diabetic patients (Evans et al. 2005) and mitigates cancer growth (Fu et al. 2017). In this study, we showed that metformin decreases the O-GlcNAc modification of AMPK, increasing AMPK activation and apoptosis in cervical cancer cells. Notably, we found that metformin increases the levels of p21 and p27, cell cycle inhibitors (Coqueret 2003), in cervical cancer cells, suggesting that metformin induces cell cycle arrest and apoptosis, confirmed by flow cytometry. Regarding metformin’s mechanism of action, several studies have shown that metformin inhibits cancer cell growth via the AMPK pathway (Shaw et al. 2005; Zakikhani et al. 2006; Queiroz et al. 2014), whereas the mechanism by which metformin regulates AMPK remains unclear. Hyper-O-GlcNAcylation promotes cell proliferation in several cancers (Kim et al. 2016; Liu et al. 2016). Indeed, we found increased levels of OGT and O-GlcNAc in HeLa cells compared to HaCaT cells, effects that were reversed by metformin. Importantly, levels of O-GlcNAcylated AMPK were increased, whereas levels of phospho-AMPK were decreased, in cervical cancer cells, effects that were reversed by metformin treatment.

O-GlcNAc modifications appear cyclical, much like phosphorylation, and functions, at least partially, via cross-talk with phosphorylation (Wang et al. 2008). As such, we found that O-GlcNAc decreased AMPK phosphorylation in cervical cancer cells. Consequently, increased AMPK activation by metformin may increase the levels of p21 and p27, consistent with results from a previous study (Queiroz et al. 2014), which, consistent with their roles as inhibitors of cyclin-CDK complexes (Coqueret 2003), may induce cell cycle arrest and apoptosis. Indeed, we found that metformin increased the levels of p21, p27, and cleaved PARP in cervical cancer cells. Further, flow cytometry results showed increased levels of apoptosis and cell cycle arrest after metformin treatment, confirming the increased levels of p21 and p21 by metformin.

Taken together, our results demonstrate that the O-GlcNAc modification of AMPK lowers AMPK activation as well as the levels of p21 and p27, and apoptosis in cervical cancer cells, effects that can be reversed by metformin treatment, strongly suggesting that metformin could be used as an antiproliferative drug in cervical cancer cells.
